# Perioperative management of drugs commonly used in patients with rheumatic diseases: a review

**DOI:** 10.6061/clinics/2017(06)09

**Published:** 2017-06

**Authors:** André Silva Franco, Leandro Ryuchi Iuamoto, Rosa Maria Rodrigues Pereira

**Affiliations:** Rheumatology Division, Faculdade de Medicina da Universidade de Sao Paulo, Sao Paulo, BR

**Keywords:** Rheumatic Diseases, Drugs, Perioperative Period, Inflammation Mediators

## Abstract

Rheumatic diseases are very prevalent, affecting about 7 million people in North America; they affect the musculoskeletal system, often with systemic involvement and potential for serious consequences and limitation on quality of life. Clinical treatment is usually long-term and includes drugs that are considered either simple or complex and are occasionally unknown to many health professionals who do not know how to manage these patients in emergency units and surgical wards. Thus, it is important for clinicians, surgeons and anesthesiologists who are involved with rheumatic patients undergoing surgery to know the basic principles of therapy and perioperative management. This study aims to do a review of the perioperative management of the most commonly used drugs in rheumatologic patients. Manuscripts used in this review were identified by surveying MEDLINE, LILACS, EMBASE, and COCHRANE databases and included studies containing i) the perioperative management of commonly used drugs in patients with rheumatic diseases: and ii) rheumatic diseases. They are didactically discussed according to the mechanism of action and pharmacokinetics; and perioperative management. In total, 259 articles related to the topic were identified. Every medical professional should be aware of the types of drugs that are appropriate for continuous use and should know the various effects of these drugs before indicating surgery or assisting a rheumatic patient postoperatively. This information could prevent possible complications that could affect a wide range of patients.

## INTRODUCTION

Rheumatic diseases are very prevalent, affecting about 7 million people in North America 1; they affect the musculoskeletal system, often with systemic involvement and potential for serious consequences and limitation on quality of life 2,3. The diagnoses of such diseases are not simple and may require additional tests and multidisciplinary approaches 4. Clinical treatment is usually long-term, using drugs that are considered either simple or complex and are occasionally unknown to many health professionals. Such a lack of understanding could lead to difficulty in managing these patients in primary care services, emergency units and surgical wards 4.

An estimated 33 million patients undergo surgical procedures each year in the United States. Serious adverse events occur in more than 1 million of these patients at an annual estimated cost of US$ 25 billion. With an aging population, it is expected that surgical indications and surgical costs will increase by 25% and 50%, respectively, and that the cost of perioperative complications will double 5.

The incidence of postoperative infections ranges from 0.5 to 6.0%, depending on the medical center, the type of surgery, and the surgical site 6. Rheumatic patients, however, are at increased risk of developing postoperative infections. Approximately 25% of all patients with rheumatoid arthritis will require surgery within the first 20 years of disease, excluding emergency procedures not related to preexisting maladies. The major complication of elective orthopedic procedures is surgical site infection, with an incidence between 2-15% and the drugs used by these patients cannot be ruled out as a contributing factors to such high rates 7–11.

Considering the epidemiology of such diseases and professional accountability for the use of prescribed drugs, it is imperative that clinicians, surgeons and anesthesiologists who are involved with rheumatic patients undergoing surgery know the basic principles of drug therapy and perioperative management 12–14.

The purpose of the present study is to evaluate the drugs that are most commonly used in patients with rheumatic diseases and to guide the perioperative management of these drugs.

## METHODS

Manuscripts used in this review were identified by surveying MEDLINE, LILACS, EMBASE, and COCHRANE databases and included studies containing i) the perioperative management of commonly used drugs in patients with rheumatic diseases: nonsteroidal anti-inflammatory drugs, glucocorticoids, disease-modifying antirheumatic drugs and biological drugs; and ii) rheumatic diseases: Behçet Syndrome, Dermatomyositis, Gout, Juvenile Arthritis, Mixed Connective Tissue Disease, Polymyalgia Rheumatica, Rheumatic Fever, Rheumatoid Arthritis, Sjogren’s Syndrome, Spondyloarthropathies, Still’s Disease, Systemic Lupus Erythematosus, Systemic Scleroderma, and Vasculitis.

There were no time or language restrictions and the last search dates back from 30 April 2016 for all databases. Articles discussing bone metabolism and cardiovascular events were excluded.

Initially, throughout all the data from the selected articles, we sought for 1 the rheumatic disease(s) each of them studied; 2 the drugs used by the patients; 3 the type of surgery; and 4 the perioperative management. Finally, we extracted the results of each article and organized them according to these aforementioned four items.

The selected items and results are discussed in this review according to the following breakdown:

- mechanism of action and pharmacokinetics of the main drugs used to control rheumatic diseases;
- perioperative management of the commonly used drugs in patients with rheumatic diseases.

## RESULTS

The search of MEDLINE, EMBASE, LILACS and COCHRANE databases provided 237 studies. After removing the duplicates, 223 remained. Of these, 18 studies were not full text; and 172 were discarded because after reviewing the abstracts these papers clearly did not meet the eligibility criteria. Twelve additional studies that met the criteria for inclusion were identified by checking the references of other relevant papers that did not meet our criteria. The full texts of the remaining 45 citations were examined in more detail. No unpublished relevant studies were obtained. 6 articles were not used in this review based on our exclusion criteria.

It is also important to note the lack of randomized, double-blind researches/trials comparing the safety of drugs in rheumatic diseases in emergency procedures and not only in elective surgeries.

In our review, we only found articles about rheumatoid arthritis, systemic lupus erythematosus and osteoarthritis, excluding any other rheumatic diseases. Regarding the treatment and perioperative management of such conditions, we found information on Nonsteroidal antiinflammatory drugs and aspirin (NSAIDs), glucocorticoids, Disease-modifying antirheumatic drugs (DMARDs) and biological drugs, presented as follows.

### Aspirin and Nonsteroidal anti-inflammatory drugs (NSAIDs)

The inhibition of COX-1 by aspirin and other NSAIDs results in increased bleeding time. This effect is lasting due to the inhibition of platelet COX and it is reversed only after drug withdrawal, which is considered to be effective after 4 to 5 half-lives. Thus, it is important to remember that depending on the average half-life of a particular NSAID, it should be suspended for hours or even days before surgical procedure.

Regarding COX-2 inhibitors, drugs that have minimal effects on platelet function 15, withdrawal during the perioperative period is not required (Table 1).

For patients who are in need of continuing aspirin treatment to manage the risk of thromboembolism / ischemia, no changes in dosage are indicated because the cardiovascular risk outweighs the intraoperative benefits of altering the drug intake 16–18. In such cases, the surgeon must be aware of this condition and be prepared for bleeding complications during the intraoperative period 19–22. Although Tytgat et al. 23 did not show differences in surgeons’ assessment of intraoperative bleeding in carotid endarterectomy among patients who stopped aspirin before surgery and those who continued aspirin intake. In fact, postoperative complications like hematomas were not significantly increased, even in surgeries including cholecystectomy, appendectomy, open or laparoscopic inguinal hernia repair, liver surgery and hip and knee arthroscopy 16,24,25. Current guideline suggests to not withdraw aspirin for secondary cardiovascular prevention before surgeries 19.

Considering rheumatic patients, the use of NSAIDs is indicated to prevent heterotopic ossification after arthroplasty, especially in patients with ankylosing spondylitis and psoriatic arthritis, for whom Indomethacin (75-100 mg/day) or Celecoxib (400 mg/day) are recommended before the fifth postoperative day, optimally within 24 to 48 hours, for 20 days after surgery 22,26,27.

### Glucocorticoid prescription due to Hypothalamic-Pituitary-Adrenal axis suppression

Hypothalamic-pituitary-adrenal axis suppression occurs in patients who are on chronic use of glucocorticoids. If the doses are above the physiologic range (10-12 mg of cortisol/day), 30 days is probably the minimal for inhibition of endogenous glucocorticoid synthesis. There is evidence that the use of 20 mg of prednisone for 5 days is already sufficient to inhibit cortisol synthesis 28. In such cases, it is necessary to supplement the glucocorticoid dose in the perioperative period due to surgical stress, even with the increased risk of infection and hindering of wound healing induced by these drugs 29,30 (Table 2).

A decade ago, high doses of corticosteroids were given to patients with adrenal insufficiency before surgery. More recent studies prefer to assess therapy length, corticosteroid dose and degree of surgical stress to prescribe the minimum amount of drug 31 (Table 2). An ACTH stimulation test could be performed during preoperative evaluation to verify the need for corticosteroid supplementation 32, although the low sensitivity observed in patients with secondary adrenal insufficiency frequently requires additional testing 33.

### Disease-modifying antirheumatic drugs

Disease-modifying antirheumatic drugs (DMARDs) are a heterogeneous group of drugs and their main benefit is to delay the progression of some rheumatologic diseases by changing its natural history. Most prospective and retrospective studies have suggested that methotrexate and other DMARDs may be continued during the perioperative period without compromising healing or increasing the risk of infection 12,34.

Hydroxychloroquine in lupus patients, reduces disease activity, cardiovascular risk, insulin resistance, thromboembolic events, infection risk and mortality 35–38, and this drug should not be discontinued during the perioperative period.

Table 3 summarizes the half-life, mechanism of action, side effects and management regarding the perioperative period.

### Biological drugs

Biological agents are newer, high cost drugs with specific mechanisms of action for each molecule - they are antibodies against a target molecule. Within rheumatology, the main agents used are those that antagonize TNF, IL-1, IL-6, CD20 and costimulatory molecules. The mechanism of action, half-life, management during the perioperative period and main side effects of each of the various biologic agents are summarized in Table 4.

For minor procedures, there is no need to interrupt most of these agents, since there is no evidence of increased risk of infection or impaired healing of the surgical site 12. However, for major surgeries, their interruption is recommended for at least twice their half-lives before surgery and may be resumed from 10 to 14 days after surgery 9,39–43, since all of these agents increase the risk of infections. Some drugs, as rituximab, an anti-CD20 monoclonal antibody that depletes populations of B lymphocytes, have a longstanding effect, beginning 2 to 3 weeks after drug introduction and lasting up to 12 months after withdrawal. Severe hypogammaglobinemia is a rare adverse effect of rituximab that could lead to infections. Serum IgG levels may be assessed prior to surgery and patients with low values (IgG < 500 mg/L) may receive intravenous immunoglobulin replacement therapy 44,45.

More studies are necessary to develop guidelines with strong evidence about the safety and management of drugs in the perioperative period in patients with rheumatic diseases. The differentiation of elective procedures and emergency surgeries would also be an important matter for the medical community to reduce infections and complications after surgery.

## CONCLUSION

Knowing the various drugs used in patients with rheumatic diseases is necessary because their side effects can modify the progression of the postoperative period. Hence, every physician, before suggesting surgical procedures or following the postoperative evolution of a rheumatic patient, should be capable of managing continuous-use drugs. The most important measures to remember are: a) Aspirin intake for secondary cardiovascular prevention should be maintained during perioperative period for most surgeries; b) NSAIDs should be suspended for hours or even days according to half-life time before surgical procedure; c) Glucocorticoid prescription must be made according to surgical stress; d) Methotrexate, hydroxychloroquine and azathioprine should be maintained during perioperative period; e) biological agents are recommended to be suspended 2 half-lives prior to surgery; f) the administration of such drugs should be restarted based on clinical status and absence of complications (infections and bleeding).

## AUTHOR CONTRIBUTIONS

Franco AS, Iuamoto LR and Pereira RM were responsible for the study design, critical analysis, manuscript drafting and approval of the final version of the manuscript. Franco AS and Iuamoto LR were responsible for the literature review. Franco AS and Pereira RM were responsible for revising the manuscript content. All the authors take responsibility for the integrity of the data analysis.

## Figures and Tables

**Table 1:** Nonsteroidal anti-inflammatory drugs.

NSAID	Half-life (hours)	Withdrawal before surgery
Naproxen	12-15	3 days
Ibuprofen	1.6-1.9	10 hours
Diclofenac	2	10 hours
Indomethacin	4.5	1 day
COX-2 inhibitor (Celecoxib)	11	maintain usual dosage

Adapted from reference 21.

**Table 2:** Glucocorticoid prescription according to surgical aggression.

Type of surgery / surgical stress	Surgical Procedures	Glucocorticoid prescription
Superficial procedure (anesthesia <1 hour)	Ophthalmologic surgeries, herniorrhaphy	Not necessary. Maintain daily dosage.
Small surgical stress	Carpal tunnel release, colonoscopy, knee arthroscopy	25 mg hydrocortisone IV or 5 mg methylprednisolone IV on the procedure day
Mild surgical stress	hip arthroplasty, knee arthroplasty, laparoscopic abdominal surgery, pulmonary biopsy	50-75 mg hydrocortisone IV or 10-15 mg methylprednisolone IV on the procedure day
Important surgical stress	bilateral hip arthroplasty, total ankle arthroplasty, spine surgery, open abdominal surgery, hysterectomy	100-150 mg hydrocortisone IV or 30 mg methylprednisolone IV on the procedure day; return to previous dosage by lowering it on the next 1 to 2 days

Adapted from references 14 and 34.

**Table 3:** DMARDs - mechanism of action, half-life and management in the perioperative period.

Drug	Half-life	Mechanism of action	Management
Methotrexate	3-10 hours	Dihydrofolate reductase inhibition	Maintain usual dosage*
Hydroxychloroquine	32-50 hours	Lysosomal membrane stabilization and reduces IL-1 and TNF synthesis	Maintain usual dosage
Leflunomide	2 weeks	Pyrimidine synthesis inhibitor - lowers B and T cell population	Withdraw 2 weeks before surgery; resume after 3 days (controversial)
Ciclosporin	5-18 hours	Inhibits T cell activation by inhibiting calcineurin – cyclophilin ligand	Withdraw 1 week before and 1 week after surgery
Azathioprine	1-3 hours	Purine synthesis inhibition – inhibits cell proliferation	Maintain usual dosage
Mycophenolate mofetil	16-18 hours	Restricts T and B cell proliferation – action upon purine-synthesising enzyme	Withdraw 1 week before surgery; resume 1 to 2 weeks after surgery

* in special situations (Chronic kidney disease, poorly controlled diabetes mellitus, etc.): methotrexate should be suspended one week before.

Adapted from references 12, 34, 39, 40 and 41.

**Table 4:** Biological agents - Half-life, mechanism of action, management during perioperative period and major side effects.

Drug	Half-life	Mechanism of action	Management	Side effects
Etanercept	3.5 – 5.5 days	Anti-TNF	Withdraw 10 days before surgery	Increased risk of infection
Adalimumab	10 – 20 days		Withdraw 30 days before surgery	
Infliximab	9.5 days		Withdraw 19 days before surgery	
Certolizumab	14 days		Withdraw 28 days before surgery	
Golimumab	14 days		Withdraw 28 days before surgery	
Abatacept	12.6 days	T cell inhibitor	Withdraw 25 days before surgery	Increased risk of infection, headache, gastrointestinal disorders
Rituximab	18 – 22 days (effects can last for months)	B cell inhibitor	Withdraw 100 days before surgery	Increased risk of infection, Stevens-Johnson syndrome, hypotension, arrhythmias
Tocilizumab	11 – 13 days	IL-6 receptor antagonist	Withdraw 26 days before surgery	Increased risk of infection, hepatotoxicity
Anakinra	4 – 6 hours	IL-1 receptor antagonist	Withdraw 1 to 2 days before surgery	Increased risk of infection, hepatotoxicity

Adapted from reference 34.
